# Drying Grapes after the Application of Different Dipping Solutions: Effects on Hormones, Minerals, Vitamins, and Antioxidant Enzymes in Gök Üzüm (*Vitis vinifera* L.) Raisins

**DOI:** 10.3390/plants11040529

**Published:** 2022-02-16

**Authors:** Nurhan Keskin, Ozkan Kaya, Fadime Ates, Metin Turan, Gastón Gutiérrez-Gamboa

**Affiliations:** 1 Faculty of Agriculture, Department of Horticulture, Van Yüzüncü Yıl University, Van 65090, Turkey; keskin@yyu.edu.tr; 2 Erzincan Horticultural Research Institute, Republic of Turkey Ministry of Agriculture and Forestry, Erzincan 24060, Turkey; 3 Manisa Viticulture Research Institute, Republic of Turkey Ministry of Agriculture and Forestry, Manisa 45125, Turkey; fadimeates2@yahoo.com; 4 Department of Genetics and Bioengineering, Faculty of Engineering, Yeditepe University, Istanbul 34755, Turkey; m-turan25@hotmail.com; 5 Escuela de Agronomía, Facultad de Ciencias, Universidad Mayor, Huechuraba 8580000, Chile

**Keywords:** autochthonous variety, dipping solution, dried grapes, drying grape processes

## Abstract

(1) Background: Raisins contain a wide range of hormones, minerals, vitamins, and antioxidant enzymes that may contribute to the health benefits of consumers. (2) Methods: The aim of this research was to compare the hormone, mineral, vitamin, and antioxidant capacities of Gök Üzüm (*Vitis vinifera* L.) raisins immersed in oak ash (OA) and potassium carbonate (PC) dipping solutions before drying. (3) Results: Abscisic acid (ABA) (5751.18–11,868.40 ng g^−1^) and riboflavin (95.17–135.54 mg 100 g^−1^) were the most abundant hormone and vitamin quantified in Gök Üzüm raisins. Glutathione S-transferase (540.07–744.85 EU gr berry^−1^), 6-glucose phosphate dehydrogenase (214.50–317.43 EU gr berry^−1^), and glucose-6-phosphate dehydrogenase (208.25–241.86 EU gr berry^−1^) enzymes presented the highest antioxidant activity in the samples. Raisins obtained after drying by immersion in OA presented higher indol-3-acetic acid (IAA), ABA, salicylic acid (SA), cytokinins (CK), and zeatin contents; glutathione reductase (GR), glucose-6-phosphate dehydrogenase (G6PD), glutathione S-transferase (GST), 6 glucose phosphate dehydrogenase (6GPD), catalase (CAT), peroxidase (POD), and superoxide dismutase (SOD) enzymatic activity; vitamin B1, B2, B6, C, and A contents; and nitrogen (N), phosphorus (P), manganese (Mn), calcium (Ca), sulfur (S), potassium (K), iron (Fe), sodium (Na), and magnesium (Mg) levels compared to the grapes dried after PC applications. (4) Conclusions: Drying Gök Üzüm grapes after the application of OA dipping solution promotes a higher content of hormones, minerals, vitamins, and antioxidant enzymes compared to PC treatments. These results could help raisin producers to make decisions when using a dipping solution to dry grapes.

## 1. Introduction

Grapevines (*Vitis vinifera* L.) are one of the most important fruit crops in terms of planted hectares and economic value in the world. Turkey is the sixth-largest grape producer in the world, accounting for an average production of 4,080,932 tons and an average surface of 440,829 ha [1]. Turkey is the second-largest raisin producer in the world, behind the United States, holding 25% of the total raisin production, and controls close to 40–45% of the traded volume, becoming the world leader in its export [2]. In Turkey, close to 35% of the grapes produced is destined for raisin production, 30% for table grapes, 25% for traditional goods (molasses), and 10% for alcoholic beverage production [1,2]. Raisins are widely consumed in the Mediterranean region due to their nutritious profile [3]. Dried grapes are rich in minerals, polyphenols, iron, potassium, magnesium, boron, calcium, and B vitamins and may have the potential to improve human health [2,3].

Raisin production is generally followed by three stages, namely pretreatment, drying, and post-drying. Drying processes considerably affect the raisin quality due to their impact on the duration of dried, sugar concentration and enzymatic activity [4]. During the drying process of grapes, there is a significant concentration in the fiber content, total carbohydrates, minerals, vitamins, fruity volatile compounds, and general antioxidant activity compared to non-dried grapes [5]. Dried grapes can be produced by traditional drying methods, such as sun drying and shade drying, or using modern drying techniques, such as shielding film drying, hot air drying, microwave drying, and vacuum drying [6]. In sun drying, the grapes are traditionally laid on racks or trays on the ground to be exposed to natural air and the sun for 2 or 3 weeks [7]. In shade drying, the grapes are dried on the rooftops of the houses without being exposed to direct sunlight, and the drying process lasts about 2–4 weeks [8]. Both shade and sun drying techniques are used to produce different raisin grape varieties in Turkey. Gök Üzüm (*V*. *vinifera* L.) is an ancient and autochthonous seedless grape variety originating from the Hadim region, Konya in Southern Turkey that is usually dried in the shade, and it retains its distinctive emerald green color when it is dried [9].

To remove the water from the grapes during the drying process, they are subjected to a pretreatment before drying [10]. In Asia Minor, dipping solutions have been used to accelerate the drying of berries in clusters. In ancient times, solutions were prepared using olive oil and wood ash, but nowadays, specially formulated potassium carbonate (K_2_CO_3_) is used, instead of wood ash with olive oil. Although a combination of ethyl esters of fatty acids and potassium carbonate is currently preferred as the active solution for most commercial cold dips, wood ash is still used as a dipping solution, because it is organic. Since enzymatic browning does not occur in the berries that dry without sunlight exposure, they retain their characteristic emerald green color. To our knowledge, there are no detailed studies that have been conducted to understand how the shade drying of grapes, after being subjected to dipping solutions, may affect the contents of minerals, vitamins, antioxidant enzymes, and hormones of Gök Üzüm raisins. Therefore, the aim of this study was to evaluate the effects of shade drying after the immersion of grapes in oak ash and potassium carbonate dipping solutions on the antioxidant enzymes, hormones, vitamins, and minerals of Gök Üzüm and, thus, to provide a theoretical basis for the selection of a suitable dipping solution for seedless grapes.

## 2. Results

### 2.1. Hormone Content in the Raisins

Seven hormones, salicylic acid (SA), gibberellic acid (GA), abscisic acid (ABA), indol-3-acetic acid (IAA), cytokinin (CK), jasmonic acid (JA), and zeatin, were determined and quantified in Gök Üzüm raisins (Table 1). ABA (5751.18–11,868.40 ng g^−1^) was the most abundant hormone analyzed in Gök Üzüm raisins, whereas GA (2.87–2.98 ng g^−1^) was found in low amounts compared to the other studied hormones. Based on the data, there were significant differences in the hormone contents between the dipping solutions applied before drying, except for the GA and JA contents. Raisins dried after an application of the oak ash (OA) treatment presented higher contents of IAA, ABA, SA, CK, and zeatin than the raisins dried after an application of potassium carbonate (PC) solution. In this fashion, the ABA content in the raisins obtained from the OA treatments was almost double that in the PS treatment (Table 1).

### 2.2. Antioxydant Enzyme Activity in the Raisins

Glutathione reductase (GR), glucose-6-phosphate dehydrogenase (G6PD), glutathione S-transferase (GST), 6 glucose phosphate dehydrogenase (6GPD), ascorbate peroxidase (APX) catalase (CAT), peroxidase (POD), and superoxide dismutase (SOD) enzymes were identified in Gök Üzüm raisins. The enzymes that presented the highest were GST (540.07–744.85 EU gr berry^−1^), followed by 6GPD (214.50–317.43 EU gr berry^−1^) and G6PD (208.25–241.86 EU gr berry^−1^) (Table 2). Significant differences were observed in the means of the treatments, except for APX enzyme activity (Table 2). Grapes dried by dipping in OA solution presented higher GR, GST, G6PD, 6GPD, CAT, POD, and SOD antioxidant enzyme activity compared to the grapes dried by dipping in PC solution. 

### 2.3. Vitamins Content in the Raisins

A total of five vitamins, vitamins A, B1, B2, B6, and C, were determined for Gök Üzüm raisins (Table 3). The most abundant vitamin was vitamin B2 (95.17–135.54 mg 100 g^−1^), followed by vitamin B6 (83.88–107.30 mg 100 g^−1^), and the least abundant vitamin was the vitamin C (12.54–17.28 mg 100 g^−1^). The results showed that the vitamins were significantly affected by the dipping solutions. In this fashion, raisins dried after the application of the OA treatment presented higher contents of vitamins A, B1, B2, B6, and C than the raisins dried after application of the potassium carbonate (PC) solution (Table 3).

### 2.4. Mineral Content in the Raisins

Twelve mineral elements, nitrogen (N), phosphorus (P), zinc (Zn), manganese (Mn), calcium (Ca), sulfur (S), potassium (K), iron (Fe), sodium (Na), boron (B), magnesium (Mg), and copper (Cu), were determined in Gök Üzüm raisins (Table 4). The results showed that the minerals were significantly affected by the dipping solutions. However, there were no significant differences in the Cu, Zn, and B contents between the dipping solutions applied before drying. In this fashion, raisins dried after the application of the OA treatment presented higher contents of N, Ca, K, Mg, Na, P, S, Mn, and Fe than the raisins dried after the application of potassium carbonate (PC) solution (Table 4). 

### 2.5. Principal Component Analysis

**Figure 1:**
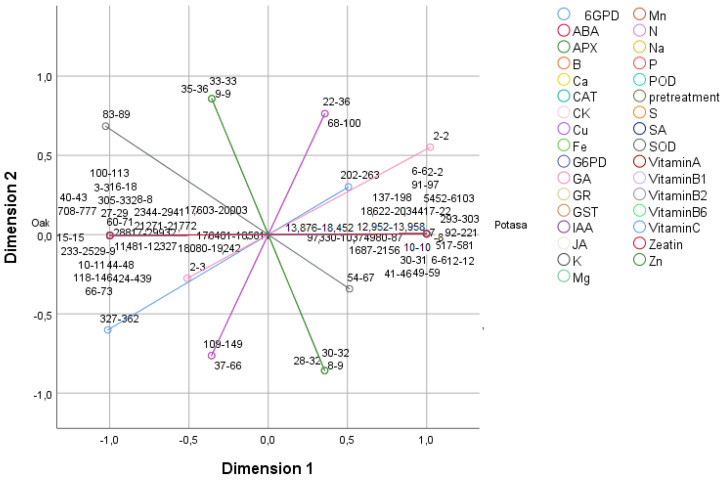
Principal component analysis (PCA). Two-dimensional (PC1 and PC2) projection of salicylic acid (SA); gibberellic acid (GA); abscisic acid (ABA); indol-3-acetic acid (IAA); cytokinin (CK); jasmonic acid (JA); zeatin; glutathione reductase (GR); glucose-6-phosphate dehydrogenase (G6PD); glutathione S-transferase (GST); 6 glucose phosphate dehydrogenase (6GPD); ascorbate peroxidase (APX) catalase (CAT); peroxidase (POD); superoxide dismutase (SOD) enzymes; vitamins A, B1, B2, B6, and C; nitrogen (N); phosphorus (P); zinc (Zn); manganese (Mn); calcium (Ca); sulfur (S); potassium (K); iron (Fe); sodium (Na); boron (B); magnesium (Mg); and copper (Cu) analyzed in the first two principal components. The values in the figure show the lower and upper data of the analyzed variables.

**Figure 2:**
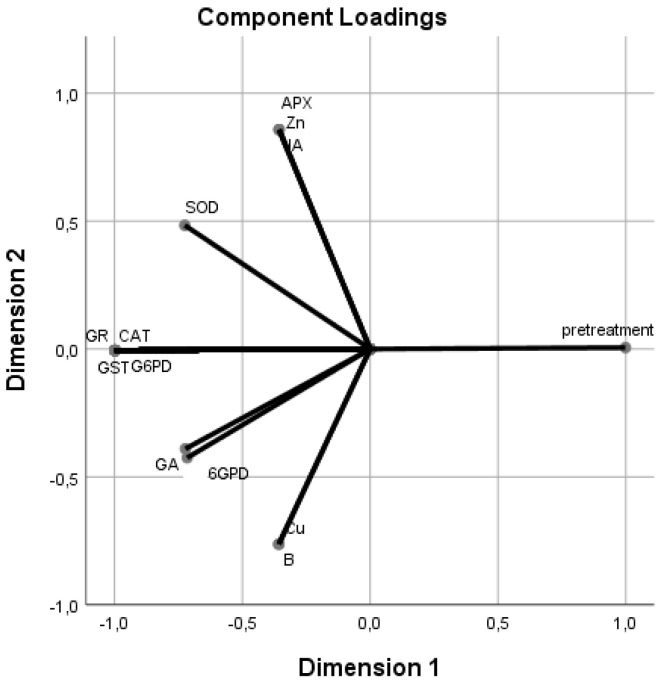
Principal component analysis (PCA). Relationships between different dipping solutions and the two-dimensional projection of salicylic acid (SA); gibberellic acid (GA); abscisic acid (ABA); indol-3-acetic acid (IAA); cytokinin (CK); jasmonic acid (JA); zeatin; glutathione reductase (GR); glucose-6-phosphate dehydrogenase (@G6PD); glutathione S-transferase (GST); ascorbate peroxidase (APX) catalase (CAT); peroxidase (POD); superoxide dismutase (SOD) enzymes; vitamins A, B1, B2, B6, and C; nitrogen (N); phosphorus (P); zinc (Zn); manganese (Mn); calcium (Ca); sulfur (S); potassium (K); iron (Fe); sodium (Na); boron (B); magnesium (Mg); and copper (Cu) in the first two main components for the Gök Üzüm grapes, which were dried after dipping in oak ash and potassium carbonate (Potas) solutions.

To classify the different treatments, assessing their influence on the hormones, enzymes, vitamins, and minerals, a principal component analysis (PCA) was performed (Figure 1 and Figure 2). Principal component 1 (PC 1) explained 82.20% of the variance, and principal component 2 (PC2) explained 11.94%, representing 94.14% of all the variance. PC 1 was closely correlated with most of the variables, except for 6 glucose phosphate dehydrogenase (6GPD), superoxide dismutase (SOD), ascorbate peroxidase (APX), gibberellic acid (GA), jasmonic acid (JA), cupper (Cu), zinc (Zn), and boron (B), while PC 2 was most closely (+) correlated with APX, JA, and Zn (Supplementary Materials). 

There was a strong correlation among glutathione reductase (GR), catalase (CAT), glutathione S-transferase (GST), and glucose-6-phosphate dehydrogenase (G6PD) enzymes, which was inversely related to the treatments applied (Figure 2). These enzymes showed a weak correlation with SOD, GA, and 6GPD. In addition, APX, Zn, and JA presented a strong correlation among them, and they were strongly inversely correlated with Cu and B and weakly correlated with SOD. Cu and B showed a strong correlation between them and were weakly correlated with GA and 6GPD.

## 3. Discussion

To our knowledge, there is no published paper that has evaluated the concentration of hormones in Gök Üzüm raisins, and we reported higher indol-3-acetic acid (IAA), abscisic acid (ABA), salicylic acid (SA), cytokinin (CK), and zeatin contents in the grapes pre-treated with oak ash than the potassium carbonate treatment. Plant hormones or phytohormones are regulators of most of the aspects of plant growth and regulate organ size, stress tolerance, pathogen defense, and reproductive development. SA has been reported to act as a regulator in the reduction/oxidation balance of plant cells, inducing morphological, physiological, and adaptive responses in plants [11]. It also participates in the activity of catalase, mitochondrial oxidase, and other related enzymes [12,13]. Thus, it is united specifically with iron-containing enzymes such as peroxidases (PODs), catalases (CATs), and aconitases [14]. Such a union negatively or positively modifies the activity of enzymes, rapidly changing the tolerance of tissues and plants to oxidative stress [15]. In our findings, the oak ash treatment maintained higher levels of SA, CAT, and POD than the potassium carbonate treatment. 

On the other hand, there is an increase in ABA content during ripening in fruits, and a treatment that retards this increase delays the induction of ripening [16]. In our study, the oak ash solution treatment probably maintained the ABA level in raisins higher than the potassium carbonate solution treatment. In addition to ABA, it has been determined by many authors that GA delays fruit ripening in some other fruits, such as peaches, tomatoes, sapotas, and mangos [17,18,19]. Our results suggest that a low GA content in raisins may be produced, because the synthesis of GA in raisins had already stopped when the ripe berries were analyzed after drying. Based on our findings, JA was the third most abundant hormone found in raisins, which is consistent with the results that the endogenous methyl jasmonate (MJ) level increases as ripening advances in mangos, apples, tomatoes, and pears [20]. As was mentioned above, to our knowledge, there are no published studies that have evaluated the content of hormones in raisins after their subjection to different dipping solutions. Grapes after harvest are treated with dipping solutions, usually an alkaline oil-in-water emulsion. This is emulsified in a solution of potassium carbonate in water, and it has been reported that ethyl esters contained within it are found to be the most effective dipping material for increasing the drying rate [21]. These compounds act on the grape skin by dissolving the waxy components, which causes a high resistance to moisture transfer, and alkaline substances such as potassium carbonate (K_2_CO_3_) facilitate the moisture transfer by breaking the skin [21]. Ribalta-Pizarro [22] reported that the hormone profile at the tissue level presented a differential accumulation of phytohormones during ripening in berry tissues, with the pulps being particularly poor in ABA, JA, and SA; seeds particularly accumulating ACC, gibberellins, and zeatin-type cytokinins; and the skin being particularly rich in auxin and active cytokinins. In this fashion, we can suggest that potassium carbonate, along with the high temperatures of the pretreatments for drying, led to an important decrease in hormones and the other studied compounds compared to the oak ash dipping solution.

As was mentioned above, there is no available information concerning the enzymatic compositions related to GR, G6PD, GST, 6GPD, APX, CAT, POD, and SOD of dried grapes after being subjected to dipping solutions. In this way, Demir et al. [23] reported that the CAT and SOD contents in raisins reached 6.05 and 3.12 U L^−1^, respectively. Our findings were quite higher than these reported values. In general, previous studies focused on the total antioxidant content and the antioxidant activity of different raisin samples [24]. The study reported a range from 7.7 to 60.9-μmol Trolox g dry weight^−1^ (DW^−1^) of antioxidant activity in these raisin samples [24]. Drying processes may affect the antioxidant capacity, and it has been determined that losses in antioxidants occur during the processing of grapes to raisins [24]. Besides, the raisins tested in this work presented total antioxidant capacities like those shown by Hogan et al. [25] in different grapevine varieties. Zhao et al. [26] reported that the antioxidants were correlated with each other, which was consistent with our results. However, the relative contribution of these antioxidant enzymes in the prevention of oxidative damage on raisins after grape drying subjected to dipping solutions has not yet been examined. Regarding our findings, the general trend observed was that the oak ash solution enhanced the antioxidant activity compared to the potassium carbonate dipping solution. Regarding other enzymes, drying can lead to a rapid rise in the sugar concentration, which inhibits the action of the polyphenol oxidase enzyme responsible for darkening in untreated fruits [27]. The enzyme is localized in the skin of the berry where drying begins, and probably, the potassium carbonate dipping solution may considerably affect their contents. In this way, Olivati et al. [28] demonstrated that drying grapes using olive oil more negatively affected the amounts of some phenolic compounds with antioxidant capacity than other pretreatments. These authors suggested that, when the cell walls are more permeable, the skins of pretreated grapes might expose the flavonols more intensely to the oxidation processes, which was favored by olive oil pretreatments. In addition, Khiari et al. [29] reported that grape berries with a truly waxy cuticle normally protect them from biotic and abiotic stresses. This layer of skin on the berries plays an important role in controlling the drying process, as waxes laid on the skin surface are both hydrophobic and serve as impermeable barriers to moisture movement along the cuticle [30]. Thus, we suggest that the skin of grapes pretreated with oak ash was exposed to lower levels of oxidative stress than the cell walls of grapes pretreated with potassium carbonate, which did not protect the raisins from the effects of reactive oxygen derivatives.

To our knowledge, this is the first report that has studied vitamin compositions of Gök Üzüm raisins obtained after the use of dipping solutions. Vitamins affect food processing due to their effects on enzymatic activities, exposure to oxygen, and storage time, which could improve the nutritional quality [31]. Based on our data, vitamin values of the samples treated with oak ash solution were higher than the samples treated with potassium carbonate solution. The USDA reported that the nutritional value of raisin reaches up to 2.3 mg of vitamin B6, 0.12 mg of vitamin E, and 0.17 mg of vitamin C in 100 g of raisins [32]. Apart from this, it was determined that the vitamin C content of Pakistani grapes reaches an average of 1.64 mg 100 g FW^−1^ [33]. It was also found that the average vitamin C content of Iranian grapes was 14.85 mg 100 g FW^−1^ for white varieties and 16.83 mg 100 g FW^−1^ for red varieties [34]. Keskin et al. [35] stated that the vitamin C in different grape varieties ranges from 6.38 mg 100 g FW^−1^ (Katıkara variety) to 12.83 mg 100 g FW^−1^ (Isabella variety). Langová et al. [36] reported that the concentration of vitamin C ranges from 0.0 to 0.58 mg 100 g^−1^ in different raisin varieties, such as Bezsemenné, Perlette, Vrboska, Beauty seedless, and Jakubské. These results are lower than those reported from the Gök Üzüm variety in this trial. The temperature of the pretreatments may play a key role in the vitamin contents by increasing moisture diffusivity. In this fashion, it was reported that there was a significant decrease of vitamin A, vitamin C, thiamine, and riboflavin when increasing the temperatures of the pretreatments to higher than 66 °C [37]. Although both treatments were carried out at the same temperature, it is possible that the oak ash treatment affected the moisture diffusivity to a lesser extent than the potassium carbonate solution, affecting less the vitamin content of the produced raisins. 

K, S, P, and Mg minerals were the most abundant elements in raisins, as has been previously stated by different authors [38,39]. Furthermore, the mean Na, Mg, Ca, F, and K contents were similar to those reported by some authors for different Tunisian and Argentine varieties [39,40]. The findings of the mineral analysis showed that the berries treated with oak ash solution had the highest amounts of N, Ca, K, Mg, Na, P, S, Mn, Cu, Fe, and Zn. This suggests that berries treated with oak ash solution lose minor amounts of their nutrient contents after drying compared to the potassium carbonate solution. Zemni et al. [41] reported that grapes dried prior to being dipped in olive oil and potassium carbonate solution presented lower contents of minerals than the grapes pre-treated with NaOH. Some studies have reported that the mineral content of raisins may affect their diffusion into the intercellular spaces, and the possible incidence of many chemical reactions under drying processing conditions [41,42].

## 4. Conclusions

The results obtained in this study showed that there were significant differences between the two dipping solutions applied before drying grapes, regarding the antioxidant enzymes, hormones, vitamins, and minerals in Gök Üzüm raisins. Abscisic acid (ABA) was the main hormone identified in the raisins, whereas gibberellic acid (GA) reached the lowest value. The enzymes glutathione S-transferase (GST), 6-glucose phosphate dehydrogenase (6GPD), and glucose-6-phosphate dehydrogenase (G6PD) showed the highest activities in Gök Üzüm raisins treated with both dipping solutions, while glutathione reductase (GR) presented the lowest activity. Vitamin B2 showed the highest concentration among the studied vitamins, whereas vitamin C had the lowest content. Drying the Gök Üzüm after being dipped in the oak ash solution promoted a higher content of most of the antioxidant enzymes, hormones, vitamins, and minerals compared to the application of potassium carbonate before drying. 

## 5. Materials and Methods

### 5.1. Plant Material and Treatments

Grapes were hand-picked from Gök Üzüm vineyards (approximately 15–20 years old) in Hadim, Konya, Turkey. Although Hadim is geographically located in the Mediterranean region, it does not fully display the characteristics of the Mediterranean climate. In this respect, it shows a transition feature between the continental climate and the Mediterranean climate. The district receives more precipitation than the regions where the continental climate is dominant. The vines were planted at a distance of 3.0 × 3.0 m, grafted onto 5-BB rootstocks, and trained with the goblet system. 

Harvested grapes were dipped in two different solutions, and subsequently, they were dried in the shade to obtain the raisins. In the pretreatments, the raisins were divided into two groups: (1) to accelerate the drying of fresh grapes and preserve their fresh green color, the clusters were treated with a dipping solution composed by potassium carbonate, reaching a temperature of 70–90 °C for 5–10 s; (2) to accelerate the drying of fresh grapes and preserve their fresh green color, the clusters were pretreated by dipping using an oak ash solution, reaching 70–90 °C for 5–10 s. To dry the grapes in the shade, the samples were placed in an airing attic (the length of the drying room was 4 to 5 m, the height was 2.5 to 4.0 m, and the width was close to 4 m with neat square vents. Grapes that dried to about 15% moisture loss over 3 to 4 weeks were removed from the attic. The study was set up in a randomized complete block design with four replicates (three bags of 300 g each were taken per rep). Thus, a total of 30 clusters (10 clusters per replication) were dried by dipping in oak ash solution and 30 clusters (10 clusters per replication) were dried by dipping in potassium solution. After drying ends, the samples were placed in polyethylene bags and stored at 20 °C until the time of analysis. The shade-dried raisins retained most of the color of the original grapes and had the most natural color.

### 5.2. Hormone Analysis in Raisins

Sample extraction was performed based on the method published by Battal and Tileklioğlu [43]. For the analysis, 80% of methanol was adjusted to −40 °C and added to 1 g of sample. The samples were homogenized with an Ultra-Turrax (IKA, Mescit Mah, Fettah Başaran, Turkey) homogenizer for 10 min, and then, it was incubated for 24 h in the dark. These samples were dried at 35 °C with evaporator pumps and dissolved using 0.1-M KH_2_PO_4_ (pH 8.0). Separation was performed using the Sep-Pak C-18 (Waters, Milford, MA, USA) cartridge, and the adsorbed hormones were transferred to amber vials using 80% methanol. Hormones were analyzed by HPLC using a Zorbax Eclipse-AAA C-18 column (Agilent, Santa Clara, CA, USA). The mobile phase for the analysis was 13% acetonitrile at pH 4.98. The flow rate was set to 1.2 mL min^−1^ and the column temperature to 25 °C. The determination of the salicylic acid (SA), gibberellic acid (GA), abscisic acid (ABA), indole-3-acetic acid (IAA), cytokinin (CK), jasmonic acid (JA), and zeatin concentrations was performed using a UV detector at 265 nm.

### 5.3. Antioxidant Enzyme Analysis in Raisins

In brief, for peroxidase (POD), superoxide dismutase (SOD), glutathione peroxidases (GPX), glucose-6-phosphate dehydrogenase (G6PD), ascorbate peroxidase (APX) glutathione S-transferase (GST), glutathione reductase (GR), and catalase (CAT) activities, the raisin were homogenized into 5 mL of 100-mM phosphate buffer (pH 7.0) containing 1% (*w*/*v*) of polyvinylpyrrolidone polymers (PVPP), and all processes proceeded at 4 °C [24]. The homogenate for the raisin samples was centrifuged at 15,000× *g* for 15 min, and the supernatant fraction of the samples was directly examined for enzyme activities. CAT activity was analyzed based at the rate of hydrogen peroxide decomposition according to the method described by Abedi and Pakniyat [44]. The CAT activity in the samples was determined by a decrease in the reaction mixture absorbance at 240 nm that was caused by adding H_2_O_2_. The reaction mixture contained 50-mM phosphate buffer (pH 7.0), 100-μL extract, and 10-mM H_2_O_2_. This reaction was run at 25 °C for 2 min after the enzyme extract was added to the samples, and the rate of decrease in the absorbance at 240 nm (E  =  39.4 mM^−1^ cm^−1^) was utilized to calculate the enzyme activity of the samples. The POD activity was measured to base its capability to turn guaiacol into tetraguaiacol at 436 nm, according to the method described by Angelini et al. [45]. The SOD activity was defined based on the determination of the inhibition in the photochemical diminution of nitro-blue tetrazolium at 560 nm, according to the methodology exposed by Abedi and Pakniyat [44]. The total SOD activity was detected by monitoring the prevention of the depletion of *p*-nitro-blue tetrazolium chloride (NBT). Two hundred microliters of the reaction mixture (50-mM phosphate buffer (pH 7.8), 50-μM riboflavin, 0.1-mM ethylenediaminetetraacetic acid (EDTA), 63-μM NBT, 13-mM methionine, and 50 μL of plant extract) were placed in the wells of a 96-well microplate under a 40 W fluorescent lamp. After 10 min of lightening, the absorbance was read at 560 nm. A nonilluminated reaction mixture, which is conducted in the same manner, was used as the blank. One unit of SOD was determined as the amount of the enzyme that produced a 50% inhibition of the NBT reduction. Raisin samples for glucose-6-phosphate dehydrogenase (G6PD, EC 1.1.1.49) and 6-phosphogluconate dehydrogenase (6PGD, EC 1.1.1.44) and glutathione reductase (GR; EC 1.8.1.7) and glutathione S-transferase (GST; EC 2.5.1.18) were washed three times with 50-mM Tris–HCl + 0.1-M Na_2_SO_4_ (pH 8.0). Each sample was homogenized by liquid nitrogen, then transferred to 100-mM PVP + 10-mM NaN_3_ + 50-mM Tris-HCl + 0.1-M Na_2_SO_4_ (pH 8.0) buffer and centrifuged at 4 °C, 15,000× g for 60 min. G6PD and 6PGD activities were determined according to the methodology described by Minucci et al. [46]. The activities of glutathione reductase (GR; EC 1.8.1.7) and glutathione S-transferase (GST; EC 2.5.1.18) were assayed by the method of Chikezie et al. [47] and Minucci et al. [46], respectively. All reactions were initiated by the addition of the enzyme solution. All enzymatic activities were determined spectrophotometrically at 25 °C using a spectrophotometer Shimadzu 1208 UV (Shimadzu Corporation, Tokyo, Japan). 

### 5.4. Vitamin Analysis in Raisins

Sliced raisins for vitamin C determination were frozen with liquid nitrogen and stored at −80 °C for analysis. Raisins were weighed during the analyses and then mixed with 2.5 mL of the extraction solution (8% acetic acid for MPA–acetic acid extraction and 0.1% oxalic acid for oxalic acid extraction and 3% MPA). This mixture was titrated with a solution of indophenol (25% DCIP and 21% NaHCO_3_ in water) until a clear but distinct rose pink color appeared. For the vitamin A analysis, raisins (0.5 g) were immersed in 20 mL of ethanol for 30 min in a water bath at 85 °C. This cooled solution was filtered through a separator funnel. Then, heptane (10 mL) was added to this solution, and it was shaken for 5 min. To allow the solution to be layered, 20 mL of 1.25% sodium sulfate was added to the tubes and shaken again for 2 min. The total tocopherols for the raisin samples were determined by its reaction with cupric ions and complexation with 2,2′-biquinoline (cuproine) according to the report by Kumar et al. [48]. Then, the solution was poured into a conical flask in which 25 mL of the extraction solution was added. A shaking water bath for 40 min at 70 °C was used to sonicate the solution. After, the samples were cooled, and they were filtered with the extraction solution to obtain a volume of 50 mL. The solution for raisins was again filtered by filter trips (0.45 µm), and a 20-µL aliquots solution was injected into the HPLC by utilizing an autosampler. For the separation of B complex vitamins of raisin samples, an analytical reversed-phase C-18 column (STR ODS-M, 150 mm × 4.6 mm I.D., 5 µm, Shimadzu Corporation, Tokyo Japan) was utilized. The mobile phase consisted of a mixture of 100-mM sodium phosphate buffer (pH 2.2) containing 0.8-mM sodium-1-octane sulfonate and acetonitrile at a 9:1 (*v*/*v*) ratio at 40 °C. The flow rate was kept constant at 0.8 mL min^−1^, and a PDA detector was used with an absorption rate of 270 nm. The detection and quantification of B vitamins was determined according to the report published by Mozumder et al. [49]. 

### 5.5. Mineral Element Analysis in Raisins

To determine the mineral elements in raisins for Gök Üzüm, the samples were ground up after drying in an oven at 68 °C for 48 h. The Kjeldahl method and a Vapodest Rapid Kjeldahl Distillation Unit (Gerhardt, Königswinter, Germany) was used to determine the total nitrogen in the raisin samples. Macroelements (K, Mg, P, Na, and Ca) and microelements (Fe, Zn, S, Cl, Cu, Mn, and B) were detected using an inductively coupled plasma spectrophotometer (Optima 2100 DV, Perkin-Elmer, Shelton, CT, USA), as was reported by AOAC et al. [50].

### 5.6. Statistical Analysis

Data were presented as the mean of three replicates and standard deviation. Kolmogorov–Smirnov normality test was performed to evaluate the normality of the variables. After this, the Mann–Whitney *U* test was used to compare the two treatments in the case where the normality assumption was not met. Principal component analysis (PCA) was also performed to determine the relationships among the hormones, antioxidant enzymes, vitamins, and macro- and micronutrients by treatment. PCA generated score vectors and loading, where the loading vectors are the correlations between the extracted principal components. The variables and score vectors are the scores of each individual case on each principal component. Since they represented the majority of the total variation, only the first two principal components were plotted, both for the score and loading plots. All the analyzed variables for raisins were standardized by a mean of zero and a variance of one prior to the PCA. The significance of the differences was determined by the LSD test (*p* ≤ 0.05), and the SPSS Statistic 21 (SPSS Inc., Chicago, IL, USA) statistical program was utilized for all statistical computations.

## Figures and Tables

**Table 1:** Hormone content (ng g^−1^ DW) of Gök Üzüm grapes dried after dipping in oak ash (OA) and potassium carbonate (PC) solutions.

Hormones	Oak Ash	Potassium Carbonate
Indol-3-acetic acid (IAA)	8.75 ± 0.28 b	6.40 ± 0.24 a
Abscisic acid (ABA)	11,868.40 ± 427.65 b	5751.18 ± 328.91 a
Gibberellic acid (GA)	2.98 ± 0.05 a	2.87 ± 0.06 a
Salicylic acid (SA)	9.45 ± 0.12 b	6.55 ± 0.04 a
Cytokinin (CK)	15.28 ± 0.17 b	10.23 ± 0.11 a
Zeatin	3.36 ± 0.09 b	2.24 ± 0.14 a
Jasmonic acid (JA)	9.23 ± 0.52 a	9.34 ± 0.21 a

Data are expressed as the mean of the data with their corresponding deviation. Different letters within a row represent significant differences (LSD test, *p* < 0.05).

**Table 2:** Antioxidant enzyme activity (EU gr berry^−1^) of Gök Üzüm grapes dried after dipping in oak ash (OA) and potassium carbonate (PC) solutions.

Antioxidant Enzymes	Oak Ash	Potassium Carbonate
Glutathione reductase (GR)	27.90 ± 1.05 b	19.69 ± 2.13 a
Glutathione S-transferase (GST)	744.85 ± 35.05 b	540.08 ± 35.63 a
Glucose-6-phosphate dehydrogenase (G6PD)	241.87 ± 9.59 b	208.25 ± 14.62 a
6 Glucose phosphate dehydrogenase (6GPD)	317.44 ± 50.26 b	214.50 ± 10.96 a
Catalase (CAT)	41.54 ± 2.02 b	32.53 ± 2.01 a
Peroxidase (POD)	60.01 ± 5.30 b	44.56 ± 2.83 a
Superoxide dismutase (SOD)	79.89 ± 11.15 b	56.23 ± 2.25 a
Ascorbate peroxidase (APX)	34.70 ± 2.09 a	31.33 ± 4.25 a

Data are expressed as the mean of the data with their corresponding deviation. Different letters within a row represent significant differences (LSD test, *p* < 0.05).

**Table 3:** Vitamin contents (mg 100 g DW^−1^) of Gök Üzüm grapes dried after dipping in oak ash (OA) and potassium carbonate (PC) solutions.

Vitamins	Oak Ash	Potassium Carbonate
Vitamin B1	70.19 ± 3.58 b	55.02 ± 5.02 a
Vitamin B2	135.54 ± 14.88 b	95.17 ± 3.26 a
Vitamin B6	107.30 ± 6.51 b	83.88 ± 0.60 a
Vitamin C	17.28 ± 1.08 b	12.54 ± 0.30 a
Vitamin A	46.64 ± 1.61 b	31.24 ± 0.26 a

Data are expressed as the mean of the data with their corresponding deviation. Different letters within a row represent significant differences (LSD test, *p* < 0.05).

**Table 4:** Macro- and micronutrient contents (mg kg^−1^) of Gök Üzüm grapes dried after dipping in oak ash (OA) and potassium carbonate (PC) solutions.

Minerals	Oak Ash	Potassium Carbonate
Nitrogen	11.31 ± 0.47 b	8.02 ± 0.07 a
Calcium	29,466.93 ± 579.74 b	19,332.86 ± 899.59 a
Potassium	180,020.47 ± 4911.81 b	101,177.28 ± 3393.71 a
Magnesium	18,628.89 ± 582.34 b	12,426.03 ± 419.78 a
Sodium	2688.42 ± 308.38 b	1893.81 ± 239.39 a
Phosphorous	21,540.92 ± 252.62 b	13,448.70 ± 498.18 a
Sulfur	18,892.61 ± 1210.07 b	13,896.70 ± 704.97 a
Manganese	320.33 ± 13.63 b	176.68 ± 33.62 a
Copper	107.15 ± 5.73 a	96.05 ± 46.29 a
Iron	430.48 ± 8.13 b	300.32 ± 5.62 a
Zinc	32.68 ± 1.85 a	33.08 ± 0.76 a
Boron	37.75 ± 1.50 a	37.86 ± 25.21 a

Data are expressed as the mean of the data with their corresponding deviation. Different letters within a row represent significant differences (LSD test, *p* < 0.05).

## Data Availability

Not applicable.

## Supplementary Materials

The following are available online at https://www.mdpi.com/article/10.3390/plants11040529/s1, CATEGORICAL PRINCIPAL COMPONENT ANALYSIS.
