# Supplementary material for: Drying Grapes after the Application of Different Dipping Solutions: Effects on Hormones, Minerals, Vitamins, and Antioxidant Enzymes in Gök Üzüm (Vitis vinifera L.) Raisins
Source: Plants (Basel). 2022 Feb 16;11(4):529. doi: 10.3390/plants11040529 (PMC8877818; doi:10.3390/plants11040529)
Supplement: Supplementary file 1 [file plants-11-00529-s001.zip › plants-1596804-supplementary.pdf]

CATEGORICAL PRINCIPAL COMPONENT ANALYSIS

In this study, categorical principal components analysis was performed to determine linear or nonlinear relationships between two pre-treatments and the studied characteristics.

Model Summary

Dimension	Cronbach's Alpha	Variance Accounted For Total (Eigenvalue)	Variance Accounted For % of Variance
1	,993	27,127	82,204
2	,769	3,939	11,936
Total	,998[a]	31,066	94,141

a. Total Cronbach's Alpha is based on the total Eigenvalue.

Plot

Category Points

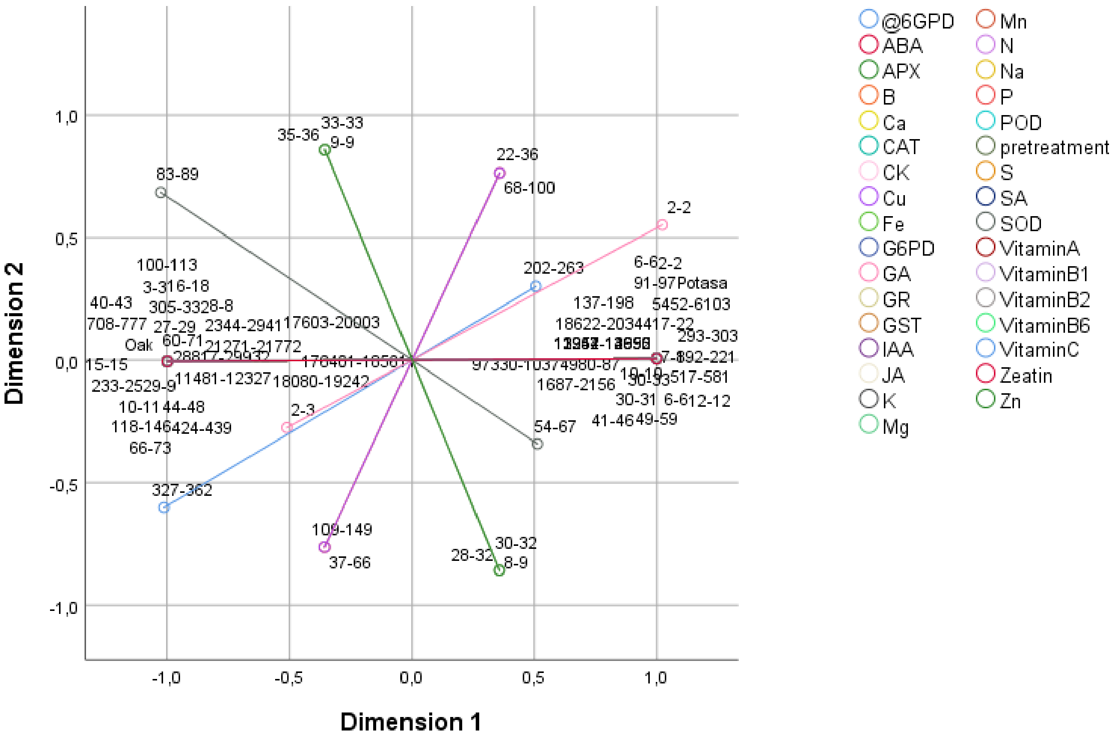

Component Loadings

Component Loadings

	Dimension	
	1	2
pretreatment	,999	,005
GR	-,999	-,005
GST	-,999	-,005
G6PD	-,999	-,005
@6GPD	-,716	-,425
CAT	-,999	-,005
POD	-,999	-,005
SOD	-,725	,484
APX	-,356	,858
IAA	-,999	-,005
ABA	-,999	-,005
GA	-,723	-,390
SA	-,999	-,005
CK	-,999	-,005
Zeatin	-,999	-,005
JA	-,356	,858
N	-,999	-,005
Ca	-,999	-,005
K	-,999	-,005
Mg	-,999	-,005
Na	-,999	-,005
P	-,999	-,005
S	-,999	-,005
Mn	-,999	-,005
Cu	-,357	-,763
Fe	-,999	-,005
Zn	-,356	,858
B	-,357	-,763
VitaminB1	-,999	-,005
VitaminB2	-,999	-,005
VitaminB6	-,999	-,005
VitaminC	-,999	-,005
VitaminA	-,999	-,005

Variable Principal Normalization.

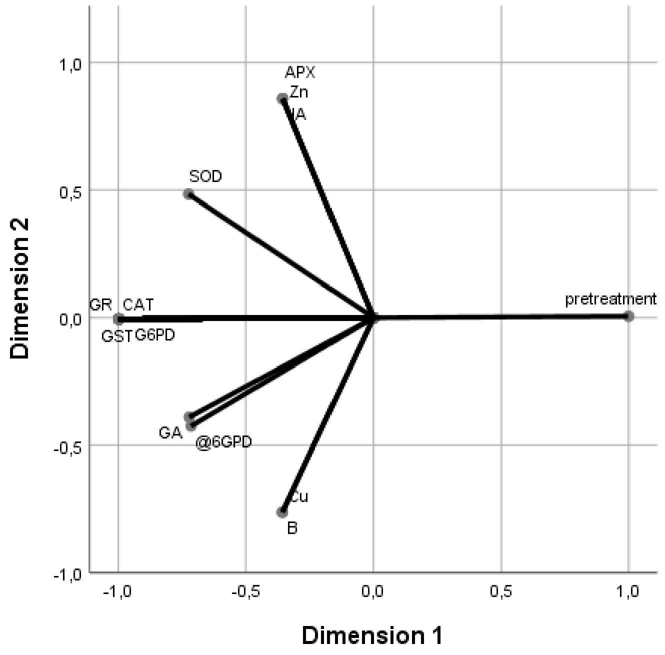

Model Summary

Dimension	Cronbach's Alpha	Variance Accounted For Total (Eigenvalue)	% of Variance
1	,968	7,183	79,809
2	,266	1,309	14,545
Total	,993[a]	8,492	94,354

a. Total Cronbach's Alpha is based on the total Eigenvalue.

Component Loadings

Component Loadings

	Dimension 1	Dimension 2
pretreatment	,994	,070
GR	-,994	-,070
GST	-,994	-,070
G6PD	-,994	-,070
@6GPD	-,713	-,500
CAT	-,994	-,070
POD	-,994	-,070
SOD	-,758	,566
APX	-,408	,842

Variable Principal Normalization.

Component Loadings

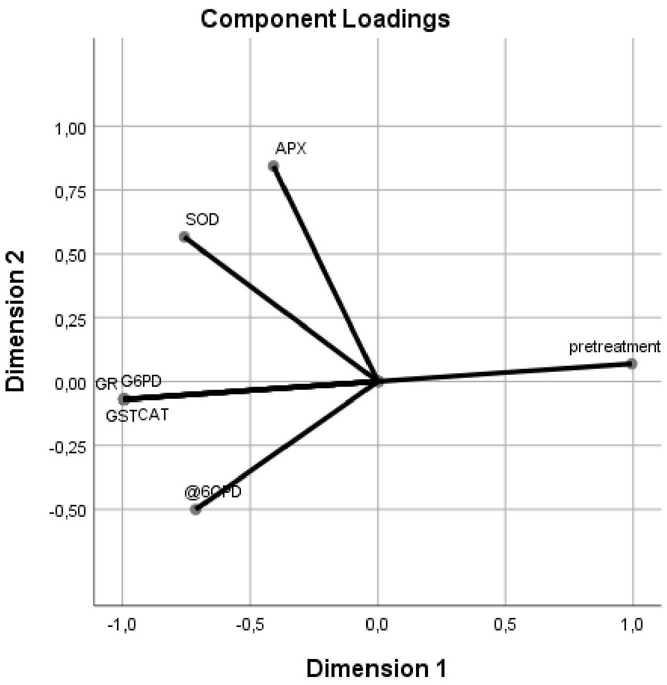

Variable Principal Normalization.

Model Summary

Dimension	Cronbach's Alpha	Variance Accounted For Total (Eigenvalue)	% of Variance
1	,971	6,649	83,113
2	,000	1,000	12,500
Total	,993[a]	7,649	95,613

a. Total Cronbach's Alpha is based on the total Eigenvalue.

Category Points

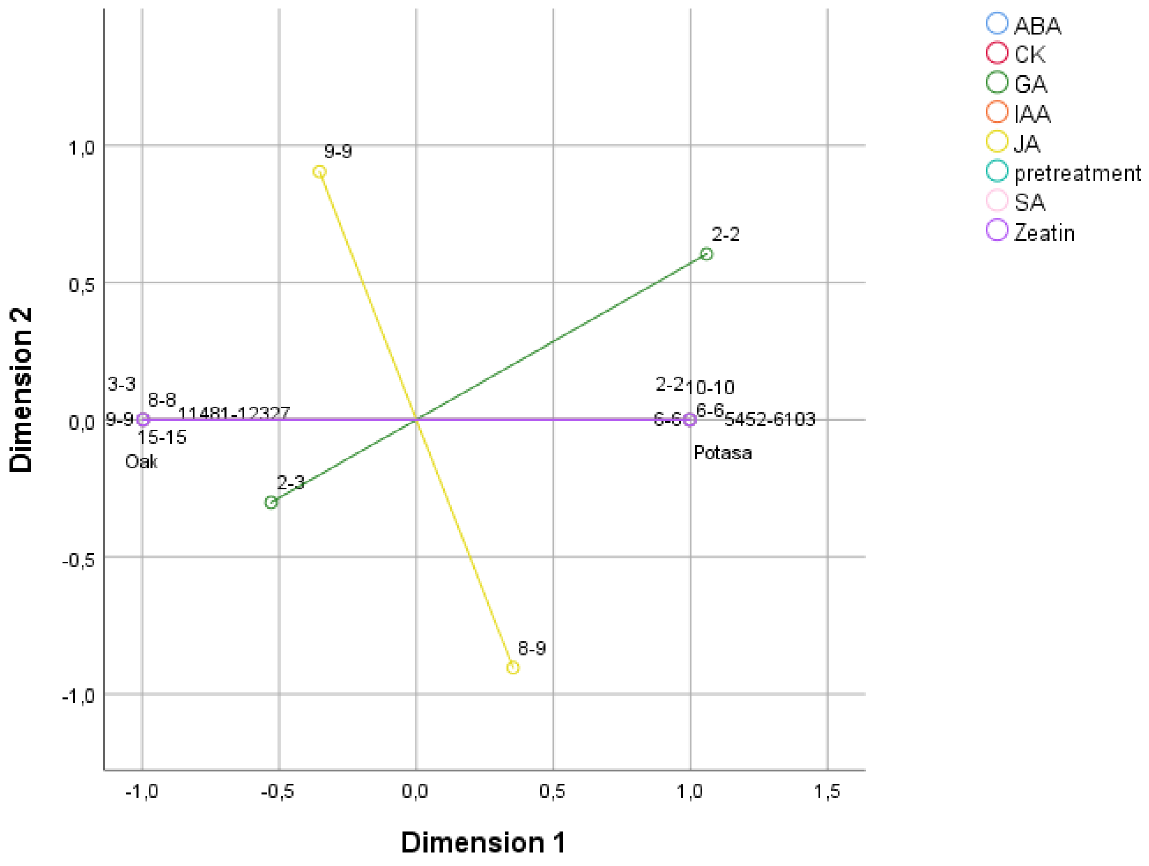

Correlations Transformed Variables

	pretreatment	IAA	ABA	GA	SA	CK	Zeatin	JA
pretreatment	1,000	-1,000	-1,000	-,707	-1,000	-1,000	-1,000	-,333
IAA	-1,000	1,000	1,000	,707	1,000	1,000	1,000	,333
ABA	-1,000	1,000	1,000	,707	1,000	1,000	1,000	,333
GA	-,707	,707	,707	1,000	,707	,707	,707	,000
SA	-1,000	1,000	1,000	,707	1,000	1,000	1,000	,333
CK	-1,000	1,000	1,000	,707	1,000	1,000	1,000	,333
Zeatin	-1,000	1,000	1,000	,707	1,000	1,000	1,000	,333
JA	-,333	,333	,333	,000	,333	,333	,333	1,000
Dimension	1	2	3	4	5	6	7	8
Eigenvalue	6,649	1,000	,351	,000	,000	,000	,000	,000

Component Loadings

	Dimension	
	1	2
pretreatment	,997	,000
IAA	-,997	,000
ABA	-,997	,000
GA	-,749	-,427
SA	-,997	,000
CK	-,997	,000
Zeatin	-,997	,000
JA	-,353	,904

Variable Principal Normalization.

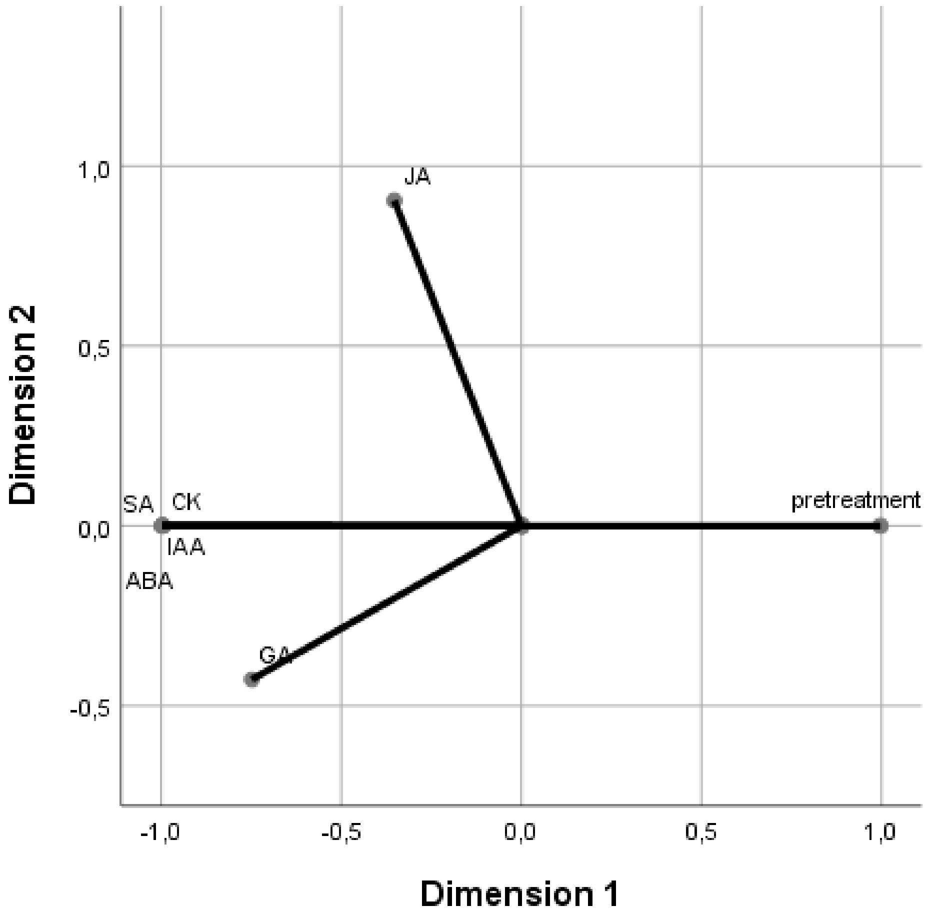

Model Summary

Dimension	Cronbach's Alpha	Variance Accounted For Total (Eigenvalue)	% of Variance
1	,979	10,366	79,741
2	,564	2,085	16,041
Total	,996[a]	12,452	95,783

a. Total Cronbach's Alpha is based on the total Eigenvalue.

Category Points

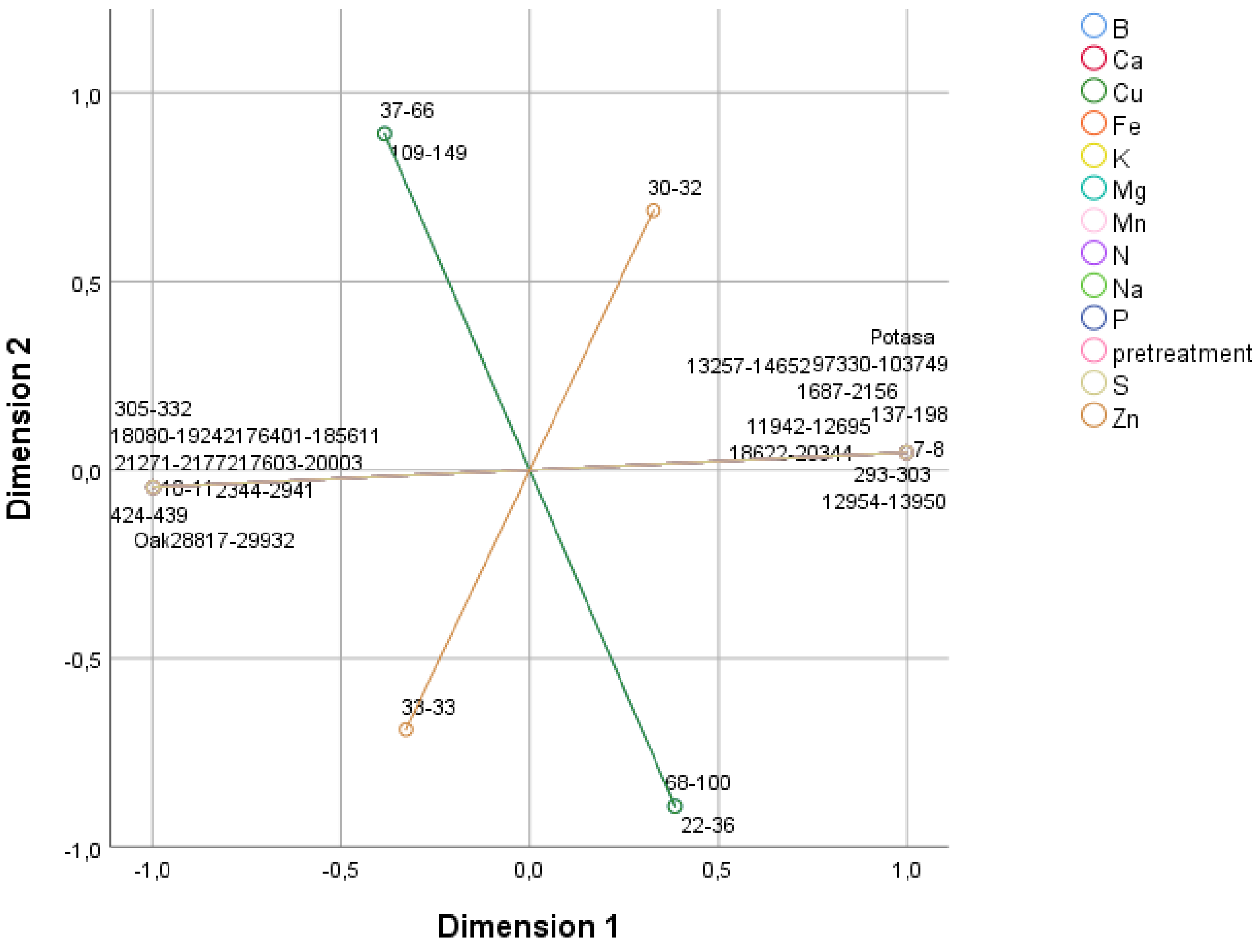

Component Loadings

	Dimension 1	Dimension 2
pretreatment	,998	,046
N	-,998	-,046
Ca	-,998	-,046
K	-,998	-,046
Mg	-,998	-,046
Na	-,998	-,046
P	-,998	-,046
S	-,998	-,046
Mn	-,998	-,046
Cu	-,385	,892
Fe	-,998	-,046
Zn	-,328	-,689
B	-,385	,892

Variable Principal Normalization.

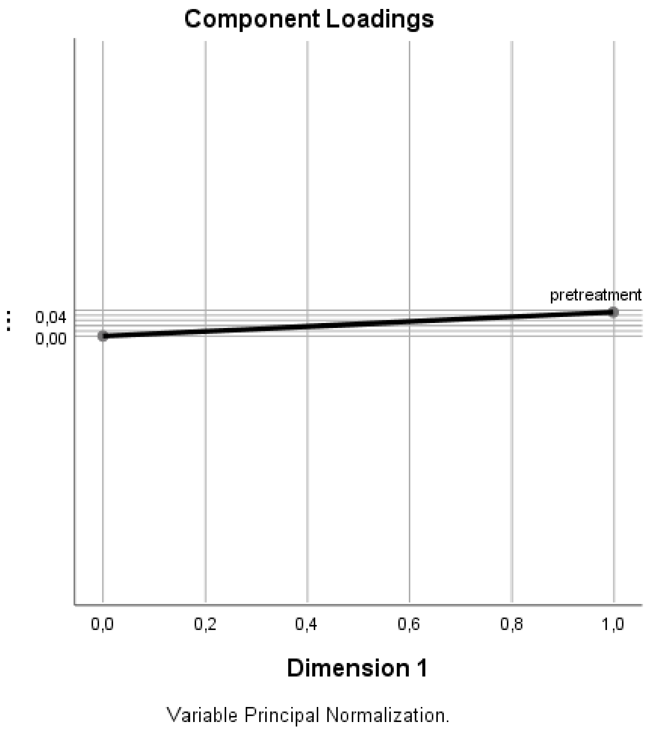

CATPCA - Principal Components Analysis for Categorical Data

Model Summary

		Variance Accounted For	
Dimension	Cronbach's Alpha	Total (Eigenvalue)	% of Variance
1	1,000	6,000	100,000
2	,000	,000	,000
Total	1,000[a]	6,000	100,000

a. Total Cronbach's Alpha is based on the total Eigenvalue.

Plot

Category Points

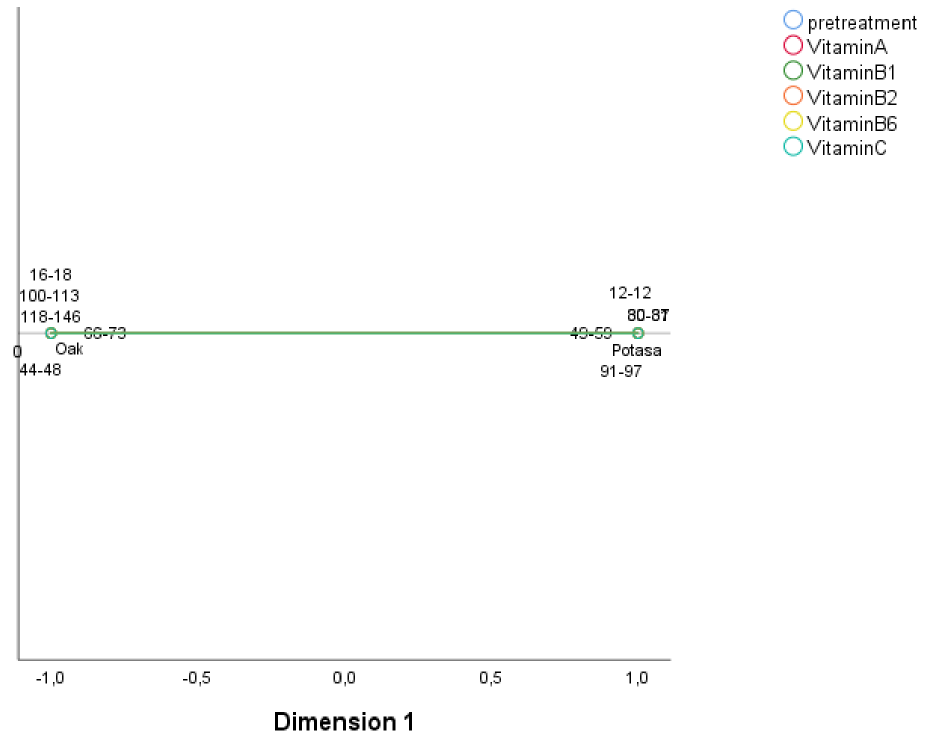

Correlations Transformed Variables

	pretreatment	VitaminB1	VitaminB2	VitaminB6	VitaminC	VitaminA
pretreatment	1,000	-1,000	-1,000	-1,000	-1,000	-1,000
VitaminB1	-1,000	1,000	1,000	1,000	1,000	1,000
VitaminB2	-1,000	1,000	1,000	1,000	1,000	1,000
VitaminB6	-1,000	1,000	1,000	1,000	1,000	1,000
VitaminC	-1,000	1,000	1,000	1,000	1,000	1,000
VitaminA	-1,000	1,000	1,000	1,000	1,000	1,000
Dimension	1	2	3	4	5	6
Eigenvalue	6,000	,000	,000	,000	,000	,000

Objects

Object Points Labeled by

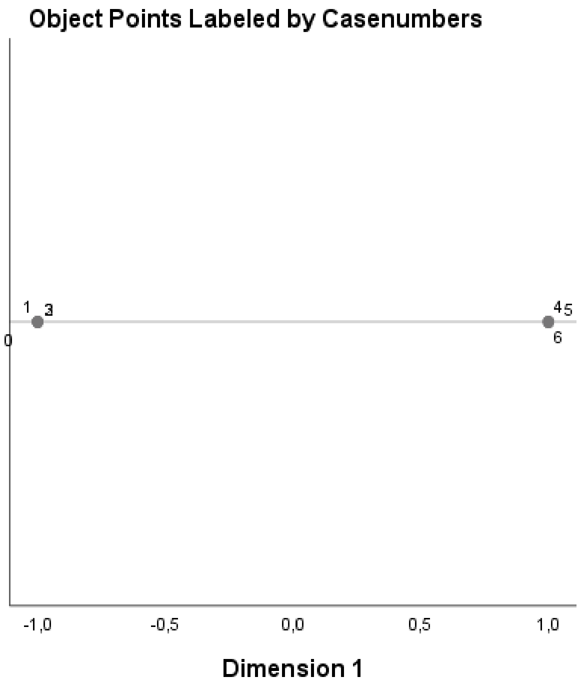

Variable Principal Normalization.

Component Loadings

Component Loadings

	Dimension	
	1	2
pretreatment	1,000	,000
VitaminB1	-1,000	,000
VitaminB2	-1,000	,000
VitaminB6	-1,000	,000
VitaminC	-1,000	,000
VitaminA	-1,000	,000

Variable Principal Normalization.

Component Loadings

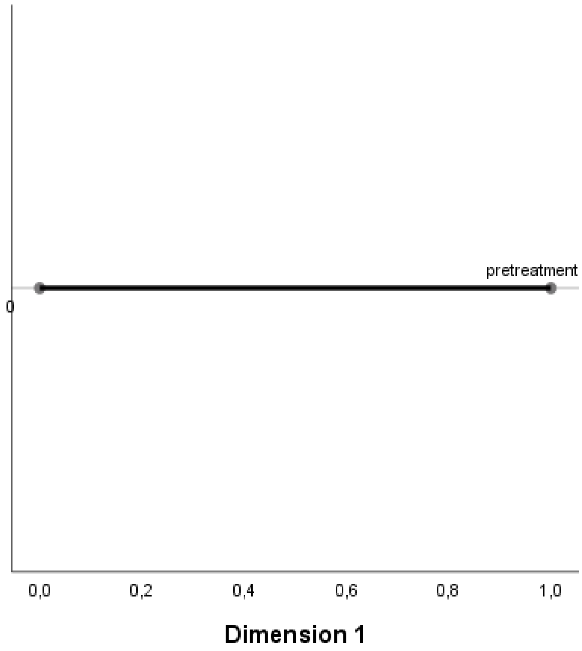

Variable Principal Normalization.
